# Barriers and Facilitators to the Use of Capnography for Respiratory Monitoring by Nurses in Phase I Post-Anesthesia Care Unit: A Scoping Review

**DOI:** 10.3390/nursrep15080292

**Published:** 2025-08-11

**Authors:** Adriana Sofia Lucas Assunção, Lara Daniela Matos Cunha

**Affiliations:** 1 Coimbra Nursing School (ESEnfC), 3004-011 Coimbra, Portugal; 2 Health Sciences Research Unit: Nursing (UICISA:E), Coimbra Nursing School (ESEnfC), 3004-011 Coimbra, Portugal

**Keywords:** capnography, nursing, post-anesthesia care unit, respiratory monitoring

## Abstract

**Background/Objectives**: Capnography monitoring in the Post-Anesthesia Care Unit (PACU) plays a crucial role in the early detection of respiratory complications, being fundamental for patient safety. It provides objective and continuous data on ventilation, enabling timely interventions to optimize health outcomes. This scoping review aims to map the available evidence regarding barriers and facilitators to the use of capnography for respiratory monitoring by nurses in the Phase I PACU. **Methods**: A scoping review was conducted following the methodology proposed by the Joanna Briggs Institute (JBI). The search was performed in the MEDLINE and CINAHL Complete databases and the Portuguese Open Access Scientific Repository (RCAAP). Studies in Portuguese, English, and Spanish were included, with no time restrictions. The search strategy combined indexing terms and natural language, adapted to each database. **Results**: Seven studies were included in the sample. The main identified barriers were a high workload, perceived lack of patient adherence, and lack of knowledge. Key facilitators included the alarm sound, patient education, anticipating patient clinical instability, increased nurse confidence, perception of enhanced safety, targeted training for nurses, continuous improvement in care delivery, effective communication and feedback, promotion of sustainable care practices, and prior knowledge and exposure. **Conclusions**: The use of capnography in the PACU allows for respiratory function assessment and the early detection of clinical events. Its implementation should be based on current scientific evidence, promoting a culture of safety and quality improvement in patient care.

## 1. Introduction

The Post-Anesthesia Care Unit (PACU) is dedicated to the continuous monitoring and care of patients following anesthetic and surgical procedures, aiming to minimize complications and ensure a safe transition to subsequent care units [1,2,3].

Most complications occur within the first five hours after anesthesia [4] and may be of surgical, anesthetic, or patient-related origin [5,6]. Phase I of the postoperative period refers to the immediate phase following surgery, during which the patient is transferred to the PACU. The primary goal is to ensure initial recovery from anesthesia and facilitate a safe transition to Phase II, thereby promoting continuity of care [7]. This phase requires close monitoring and specialized interventions to maintain hemodynamic stability, prevent complications, and ensure patient safety [7].

In the PACU, nurses hold a critical role in managing the immediate recovery of patients following anesthesia. They are responsible for continuous and autonomous clinical monitoring, the early identification of complications, and rapid, informed decision-making, supported by specialized knowledge in perioperative nursing [8].

This professional practice is regulated by national and international frameworks that acknowledge the complexity and specificity of care in this context. In Portugal, the Portuguese Nursing Council recognizes the specialty of Nursing Care to a Person in Perioperative Situations, which focuses on the care of individuals and families undergoing surgical and anesthetic experiences. This includes clinical surveillance, risk management, complication prevention, and the promotion of safe and efficient care [9].

The use of advanced monitoring technologies, such as capnography, highlights the need for specialized clinical competencies, enabling nurses to detect early ventilatory changes and respond promptly. The Association of periOperative Registered Nurses (AORN) reinforces this autonomous and specialized role of nurses in the Phase I PACU, emphasizing their contribution to patient safety and effective recovery [10]. Evidence-based practice, combined with clinical reasoning and effective interdisciplinary collaboration, is essential to ensure timely and person-centered care responses.

Common respiratory complications during this phase include post-extubation hypoxemia, bronchospasm, atelectasis, acute respiratory failure, pneumothorax, pleural effusion, exacerbation of underlying diseases, and airway obstruction [11,12].

Capnography is a non-invasive, continuous technique that assesses the effectiveness of ventilation [13] and enables the early detection of respiratory deterioration, being more sensitive than pulse oximetry in identifying adverse events [13,14].

Studies show that capnography can detect respiratory adverse events 8 to 11 min earlier than conventional monitoring [15], identify more episodes of postoperative respiratory depression compared to oximetry, and is six times more accurate than other methods [16].

While not universally mandatory, the use of capnography is strongly recommended for patients receiving opioid analgesia, individuals with obstructive sleep apnea (OSA), and those requiring supplemental oxygen therapy, due to their increased risk for respiratory complications [17,18,19,20].

Despite its clinical relevance, capnography is not yet widely implemented in the PACU [21,22]. Identifying barriers and facilitators can support a more effective and sustained implementation of this monitoring tool in clinical practice, ultimately enhancing patient safety and quality of care.

Despite its demonstrated clinical relevance, the use of capnography in the Phase I PACU remains inconsistent. Observations from clinical practice and the evidence found indicate that even when the equipment is available and nurses recognize its benefits, respiratory monitoring using capnography is not systematically implemented. Understanding the factors that hinder or facilitate its adoption may contribute to a more sustained and effective integration into clinical practice.

A preliminary search conducted between October and December 2023 in the databases MEDLINE (via PubMed), CINAHL Complete (via EBSCOhost), JBI Database of Systematic Reviews, Cochrane Database of Systematic Reviews, and PROSPERO did not identify any existing or ongoing systematic or scoping reviews on this specific topic, reinforcing the relevance and originality of the present review.

This scoping review aims to map the evidence on barriers and facilitators to the use of capnography for respiratory monitoring by nurses in the Phase I PACU.

## 2. Methods

This scoping review was conducted in accordance with the methodological guidelines established by the Joanna Briggs Institute (JBI) [23]. The review team opted not to register the protocol in advance.

This scoping review was conducted in accordance with the Preferred Reporting Items for Systematic Reviews and Meta-Analyses Extension for Scoping Reviews (PRISMA-ScR) reporting guidelines [24].

### 2.1. Identifying the Research Question

Following the JBI-recommended Population/Concept/Context (PCC) framework, the review question was: “*What are the barriers and facilitators to the use of capnography for respiratory monitoring by nurses in Phase I PACU?*”

The PCC elements:

- Population: Nurses
- Concept: Barriers and facilitators to the use of capnography for respiratory monitoring
- Context: Phase I PACU

### 2.2. Identifying Relevant Studies

The literature search was conducted in MEDLINE (via PubMed), CINAHL Complete (via EBSCOhost), and the Portuguese Open Access Scientific Repository (RCAAP). Search terms included: nurs*, capno*, “respiratory monitoring”, “carbon dioxide”, “end tidal carbon dioxide”, “respiratory assessment”, “respiratory complications”, “postanesthesia”, “recovery”, “postoperative”, PACU, “post anesthesia care unit”, and “immediate postoperative”. These were combined with specific Medical Subject Headings (MeSH) and CINAHL Subject Headings for each respective database, as detailed in Appendix A (see Table A1, Table A2 and Table A3). The search was conducted in January 2024 with no time restrictions.

### 2.3. Study Selection

Inclusion and exclusion criteria were based on population, concept, and context. Studies that included nurses—irrespective of their area of specialization—or multidisciplinary teams involving nurses were considered for inclusion. To be eligible, studies were required to focus on respiratory monitoring using capnography, addressing relevant barriers, facilitators, challenges, and limiting factors. Only studies conducted in perioperative settings involving adult patients (aged 18 and older) within Phase I PACU environments, defined as the immediate postoperative period, were included. Studies limited to preoperative or exclusively intraoperative contexts were excluded.

Only studies published in Portuguese, English, or Spanish were included, as these were the languages spoken by the reviewers. No time restrictions were applied.

All results from the different databases were exported to Rayyan QCRI (Qatar Computing Research Institute), where duplicate records were removed. A blinded and independent screening of titles and abstracts was conducted in duplicate by two reviewers (A.A. and L.C.) on 18 January 2024 to apply the inclusion criteria.

Disagreements between the reviewers were resolved through consultation with a third reviewer, ensuring impartiality and consensus. The third reviewer was not directly involved in the development of the manuscript and therefore is not listed as an author. However, they exclusively contributed by mediating decisions during the study selection process. The reviewers have academic training and clinical experience in post-anesthesia care, ensuring familiarity with the topic under study, and declared no conflicts of interest related to the included studies or the review topic.

The full texts of the potentially eligible studies were obtained and analyzed in detail. Reference lists of included studies were also reviewed to identify the additional relevant literature. Although a formal blinded review of full texts was not implemented, the combination of an independent duplicate review with mediation by a third reviewer was adopted to minimize the risk of bias and increase the reliability of the process.

### 2.4. Data Extraction and Comprehensive Analysis

Data from the included studies were independently extracted by two reviewers using a customized data extraction matrix, which included the following: author, year of publication, title, country, study design, objectives, participants, setting, and main findings related to the barriers and facilitators of capnography use.

In this study, barriers are defined as factors that limit, hinder, or negatively affect the effective use of capnography for respiratory monitoring by nurses in Phase I of the PACU. Facilitators are elements that help promote or facilitate the use of capnography. Barriers and facilitators are essential factors influencing the implementation process of healthcare practices [25].

### 2.5. Reporting the Results

The results are summarized and presented in tabular form aligned with the objective of this scoping review. A narrative summary accompanies the tables and describes how the findings relate to the review’s objective and question.

## 3. Results

**Figure 1:**
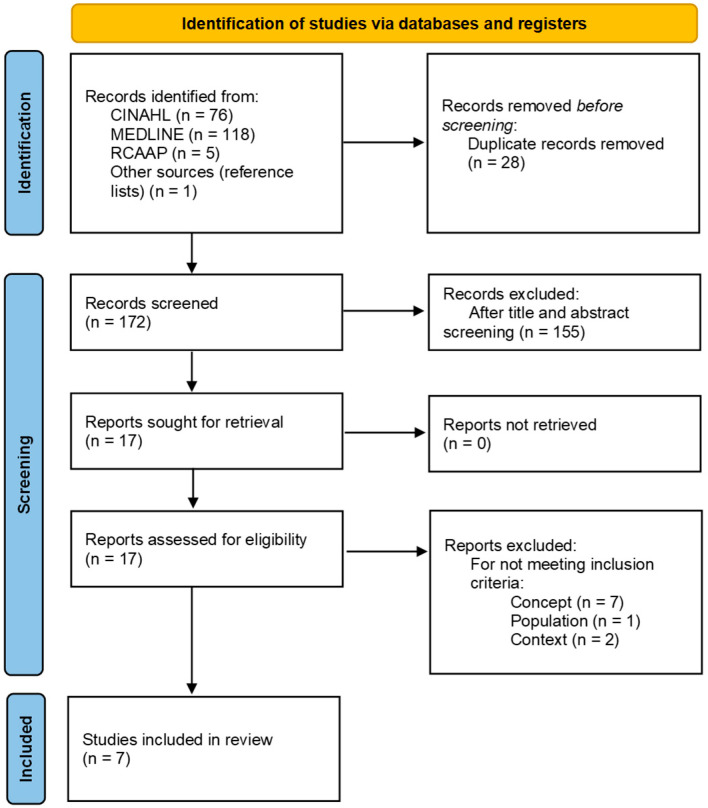
The PRISMA Flow Diagram for study selection.

The results of the search and article selection process are presented using the PRISMA (Preferred Reporting Items for Systematic Reviews and Meta-Analyses) flowchart, adapted for scoping reviews (Figure 1).

The search process initially identified 200 articles. Of these, 28 were excluded as duplicates, leaving 172 articles for title and abstract screening. From these, 155 were excluded. After the full-text reading of the remaining 17 eligible articles and applying the inclusion criteria, seven articles were included in the review.

Table A4, presented in Appendix B, contains the data extracted from the studies included in this scoping review, based on the customized data extraction matrix developed for this purpose. Table 1 provides a summary of the barriers and facilitators identified in each study regarding the use of capnography for respiratory monitoring by nurses in the Phase I PACU.

The analysis of the seven studies included in this scoping review allowed the identification of a set of barriers and facilitators to the use of capnography for respiratory monitoring by nurses in Phase I of the PACU.

Among the identified barriers, a high workload stands out, as nurses reported difficulties integrating capnography into daily practice due to competing demands, especially in the demanding PACU context, which can limit time and attention devoted to capnography monitoring [26]. Additionally, the perceived lack of patient adherence, caused by patients’ difficulties in complying with device use (e.g., keeping nasal cannulas in place) may discourage consistent monitoring [26,28,29]. Finally, lack of knowledge, manifested as limited understanding of the interpretation and application of capnography by nurses, constitutes a significant obstacle to its effective use [28,29].

Among the facilitators for the use of capnography, the alarm sound plays a key role by enhancing vigilance and prompting timely interventions [26]. Patient education promotes adherence to capnography through patient instruction supporting successful monitoring and improving patients’ compliance with device use [26,28]. Nurses who anticipate patient clinical instability by using capnography to proactively identify deterioration perceive it as a valuable clinical tool [26,27,28,30]. Increased nurse confidence, gained through familiarity with the technology, boosts willingness to apply it in clinical practice [27,30]. The perception of enhanced safety leads nurses to associate capnography with increased patient safety [21,27,28,30,31]. Targeted nurse training equips professionals with the skills to interpret capnographic data and respond appropriately, with nurses receiving specific training demonstrating greater adherence to its use [21,27,29,30,31].

The integration of capnography supports continuous improvement in care delivery, aligning with the broader goals of care optimization and innovation [30]. Effective communication and feedback, including sharing monitoring data and patient outcomes, strengthen interprofessional collaboration and reinforce capnography’s integration into practice [30]. Additionally, capnography promotes sustainable practices by supporting efficiency and resource stewardship in clinical settings, enabling early interventions, minimizing complications, and optimizing resources [30]. Finally, prior knowledge and exposure to capnography positively influence its adoption and routine use [31].

Table 2 provides a summary of the identified categories and the contributions of the seven included studies.

## 4. Discussion

This review synthesized current evidence from selected sources on the use of capnography by nurses in the Phase I PACU, identifying key barriers and facilitators that influence its implementation for respiratory monitoring. The findings highlight a complex interplay between individual, clinical, and organizational factors that shape nursing practice. While capnography is widely recognized for enhancing patient safety and enabling the early detection of respiratory compromise, its consistent application is influenced by nurses’ knowledge, workload, perceived patient cooperation, and institutional support. These insights underscore the need for targeted interventions—such as structured training, communication strategies, and system-level support—to optimize the use of capnography and strengthen postoperative care quality.

The immediate postoperative period is a critical phase during which nurses must remain vigilant for respiratory events and early signs of respiratory depression such as tachycardia, drowsiness, or altered consciousness [32]. While opioid administration is a common cause of respiratory depression, other factors such as OSA also contribute, making capnography a more sensitive and accurate monitoring tool compared to pulse oximetry or respiratory rate evaluation [26,27]. Continuous, objective capnographic monitoring enables nurses to anticipate respiratory instability, thus preventing complications through early intervention [26,27,28,30]. The ongoing analysis of capnography data supports dynamic clinical decision-making, reinforcing the need for care plans to be regularly updated with timely, evidence-based interventions [21,29].

A prominent barrier identified is the lack of knowledge and training among PACU nurses regarding capnography interpretation and application. Many nurses report unfamiliarity with the technology and its clinical significance, which limits its integration into routine practice [29]. Consequently, structured and continuous education is essential for building the competencies required to interpret capnography accurately and make informed clinical decisions [21,26,30]. Targeted training not only enhances technical skills but also increases nurses’ confidence, which is pivotal for fostering the critical thinking and clinical reasoning necessary for effective respiratory monitoring [27,30,31]. The implementation of standardized protocols and guidelines can further support nurses, providing a consistent framework for capnography use and facilitating evidence-based practice [21,33].

Beyond individual competencies, the role of healthcare organizations, leadership, and institutional culture is fundamental in supporting capnography adoption. Organizational support through policy development, resource allocation, and integration of monitoring protocols creates an enabling environment for nurses to utilize capnography effectively [34]. Leadership commitment is crucial for promoting a safety culture that prioritizes respiratory monitoring and encourages the uptake of new technologies. The presence of clinical champions and continuous quality improvement initiatives, such as audit and feedback cycles, fosters accountability and reinforces practice standards [27,30]. Effective communication within the healthcare team ensures shared understanding and alignment in respiratory monitoring practices, which is vital for cohesive and timely patient care.

The PACU is a multidisciplinary environment where collaboration between nurses, anesthesiologists, respiratory therapists, and other professionals influences the implementation of capnography. Interprofessional dynamics can either facilitate or hinder the consistent use of capnography, depending on the clarity of roles, mutual respect, and communication pathways. Encouraging collaborative decision-making and open dialog enhances the acceptance of capnography data in clinical judgments and fosters coordinated responses to respiratory deterioration [27,30]. Feedback mechanisms that include the entire care team contribute to a shared responsibility for patient safety and promote continuous learning.

Patient-related barriers such as non-adherence due to discomfort from nasal cannulas or distress caused by alarm sounds present challenges to effective monitoring [26,27,29]. Patient education is therefore a critical facilitator, helping patients understand the importance of capnography and improving cooperation with device use [26]. Alarm fatigue among healthcare professionals is another significant concern; excessive or false alarms may desensitize nurses and delay responses, thereby compromising patient safety [33,34]. Strategies to optimize alarm settings and minimize unnecessary alerts, coupled with ongoing staff training, are essential to mitigate this issue.

The review underscores the necessity of developing specific competencies in capnography interpretation, clinical decision-making, and patient communication. Nursing education programs and continuing professional development should incorporate these components to prepare nurses for enhanced respiratory monitoring roles. Healthcare organizations must actively foster a culture that values innovation, supports training initiatives, and facilitates interprofessional collaboration to embed capnography into standard PACU care.

This review presents several strengths. It is, to our knowledge, the first scoping review to comprehensively map the barriers and facilitators to capnography use by nurses in the Phase I PACU. The review also synthesized findings across different healthcare systems and contexts, enhancing the transferability of the results. Furthermore, by categorizing barriers and facilitators at individual, clinical, and organizational levels, the review provides a structured framework that can inform targeted interventions in nursing practice, education, and policy.

While this review offers several contributions, certain limitations must also be acknowledged. The primary limitations of this scoping review include the limited number of databases consulted and the language restrictions, which may have impacted the breadth of the evidence identified. These limitations should be taken into account when interpreting the findings.

Future research should explore the impact of leadership styles, institutional policies, and team dynamics on capnography implementation. Additionally, evaluating the effectiveness of tailored educational interventions and protocol-driven care pathways will provide further evidence to optimize respiratory monitoring strategies.

## 5. Conclusions

This scoping review highlights the critical role of integrating capnography into the Phase I PACU for respiratory monitoring. By identifying key barriers—such as limited knowledge, a high workload, and perceived lack of patient adherence, and facilitators—including nurse training, prior experience, and the perception of increased safety, it offers practical insights to enhance implementation.

Capnography, when used alongside other monitoring methods, is recognized as a valuable tool for the early detection of respiratory complications and for strengthening patient safety. Institutional support through effective communication, continuous training, and quality improvement initiatives remains essential.

These findings can inform nursing practice and health policy development, although the review’s scope was limited by the number of databases searched and language restrictions.

## Figures and Tables

**Table 1:** Data extraction from the studies included in the scoping review based on the PCC.

Reference Number	First Author’s Surname, Year of Publication	Population	Context	Concept (Barriers and Facilitators to the Use of Capnography for Respiratory Monitoring by Nurses in Phase I PACU)
[26]	Hutchison et al. (2008)	54 adult patients following orthopedic surgery, monitored for respiratory depression using capnography by nurses	PACU and general nursing care unit	**Barriers:** - Capnography requires more time from professionals to be implemented; - Nurses perceived greater difficulty in patient adherence due to limitations in daily activities. **Facilitators:** - Educating patients to facilitate their adherence to monitoring; - The alarm produced by the capnography monitor led to a quicker response from nurses; - Allowed early identification of changes in respiratory function.
[27]	McCarter et al. (2008)	634 adult postoperative patients receiving Patient Controlled-Analgesia (PCA), monitored with capnography by nurses	Postoperative period (Phase I and Phase II)	**Facilitators:** - Increased nurse awareness of respiratory changes; - Increased effectiveness of nursing interventions to minimize respiratory discomfort related to PCA; - Increased nurse confidence due to effective monitoring; - Quick response from the team to prevent serious consequences; - Increased perceived patient safety during this period.
[28]	Lakdawala et al. (2017)	161 adult neuro-surgical patients screened for OSA using STOP-Bang, monitored with capnography by nurses	Preoperative unit, PACU, and neuro-surgery unit	**Barriers:** - Nurses observed greater patient resistance to adherence to capnography devices; - Nurses reported the need for increased knowledge on the use of capnography in patients with OSA. **Facilitators:** - Capnography proved to identify early signs of respiratory depression, allowing for quick intervention; - Need to educate patients on the importance of using the device to ensure safety and promote adherence.
[29]	Jungquist et al. (2019)	60 adult patients in PACU after spine, neck, hip, or knee surgery, monitored by nurses for opioid-induced respiratory depression	PACU	**Barriers:** - Nurses’ perception of patient non-adherence, related to discomfort and mask removal for nursing care. - Need for nurse training due to limited knowledge of the technique and indications for capnography.
[30]	Scully (2019)	314 adult patients diagnosed with OSA, monitored by multidisciplinary team	PACU	**Facilitators:** - Training for professionals (introduction to capnography and monitoring); - Increased confidence in using capnography helped stimulate critical thinking (by applying it to other patients beyond the OSA population); - Improvement in care quality and patient safety drive change; - Regular communication of results (via audit) motivated the team and promoted the implementation process; - Promoting sustainability by involving training and raising nurse awareness to provide better care, integrating capnography monitoring into clinical practice; - Allowed the nurse to intervene early to prevent respiratory complications.
[31]	Atherton et al. (2022)	176 nurses	PACU	**Facilitators:** - The educational program increased nurses’ confidence and competence in using End-tidal Carbon Dioxide Concentration (etCO2) and transcutaneous carbon dioxide monitoring; - Prior knowledge and training with etCO2 monitoring.
[21]	Potvin et al. (2022)	52 adult patients with endotracheal tube or laryngeal mask, monitored by nurses	PACU	**Facilitators:** - Nurse training facilitated the accurate interpretation and the layout of monitoring systems; - Training should be accompanied by a standardized protocol; - Standard monitoring, together with capnography, can improve patient safety by allowing early detection of respiratory complications.

**Table 2:** Summary of the barriers and facilitators to the use of capnography for respiratory monitoring by nurses in the Phase I PACU.

		Hutchison et al. (2008) [26]	McCarter et al. (2008) [27]	Lakdawala et al. (2017) [28]	Jungquist et al. (2019) [29]	Scully et al. (2019) [30]	Atherton et al. (2022) [31]	Potvin et al. (2022) [21]
Barriers	High workload	X						
	Perceived lack of patient adherence	X		X	X			
	Lack of knowledge			X	X			
Facilitators	Alarm sound	X						
	Patient education	X		X				
	Anticipating patient clinical instability	X	X	X		X		
	Increased nurse confidence		X			X		
	Perception of enhanced safety		X	X		X	X	X
	Targeted nurse training		X		X	X	X	X
	Continuous improvement in care delivery					X		
	Effective communication and feedback					X		
	Promotion of sustainable practices					X		
	Prior knowledge and exposure						X	

## Data Availability

Data are contained within the article.

## Public Involvement Statement

No public involvement in any aspect of this research.

## Guidelines and Standards Statement

This manuscript was drafted in accordance with the PRISMA 2020 (Preferred Reporting Items for Systematic reviews and Meta-Analyses) guideline for systematic review research.

## Abbreviations

The following abbreviations are used in this manuscript:

PACU	Post-Anesthesia Care Unit
AORN	Association of periOperative Registered Nurses
JBI	Joanna Briggs Institute
RCAAP	Open Access Scientific Repositories of Portugal
OSA	Obstructive Sleep Apnea
PRISMA-ScR	Preferred Reporting Items for Systematic Reviews and Meta-Analyses extension for Scoping Reviews
PCC	Population/Concept/Context
QCRI	Qatar Computing Research Institute
PRISMA	Preferred Reporting Items for Systematic Reviews e Meta-Analyses
PCA	Patient Controlled-Analgesia
etCO_2_	End-tidal Carbon Dioxide Concentration
USA	United States of America

## Appendix A. Search Strategy

**Table A1:** Search strategy—MEDLINE (PubMed)—search performed on 12 January 2024.

Search	Search Terms	Results
#1	nurs* [Title/Abstract]	546,738
#2	“Postanesthesia Nursing” [MeSH Terms] OR “Nurses” [MeSH Terms] OR “Nursing” [MeSH Terms]	338,351
#3	“nurs*” [Title/Abstract] OR “Postanesthesia Nursing” [MeSH Terms] OR “Nurses” [MeSH Terms] OR “Nursing” [MeSH Terms]	696,705
#4	“capno*” [Title/Abstract] OR “respiratory monitoring” [Title/Abstract] OR “carbon dioxide” [Title/Abstract] OR “end tidal carbon dioxide” [Title/Abstract] OR “respiratory assessment” [Title/Abstract] OR “respiratory complications” [Title/Abstract]	76,533
#5	“Capnography” [MeSH Terms] OR “blood gas monitoring, transcutaneous” [MeSH Terms] OR “Carbon Dioxide” [MeSH Terms] OR “Pulmonary Ventilation” [MeSH Terms] OR “signs and symptoms, respiratory” [MeSH Terms]	322,554
#6	“capno*” [Title/Abstract] OR “respiratory monitoring” [Title/Abstract] OR “Carbon Dioxide” [Title/Abstract] OR “end tidal carbon dioxide” [Title/Abstract] OR “respiratory assessment” [Title/Abstract] OR “respiratory complications” [Title/Abstract] OR “Capnography” [MeSH Terms] OR “blood gas monitoring, transcutaneous” [MeSH Terms] OR “Carbon Dioxide” [MeSH Terms] OR “Pulmonary Ventilation” [MeSH Terms] OR “signs and symptoms, respiratory” [MeSH Terms]	366,211
#7	“Postanesthesia” [Title/Abstract] OR “Recovery” [Title/Abstract] OR “Postoperative” [Title/Abstract] OR “PACU” [Title/Abstract] OR “Post anesthesia care unit” [Title/Abstract] OR “Immediate postoperative” [Title/Abstract]	1,188,357
#8	“Postoperative Period” [MeSH Terms] OR “Postoperative Care” [MeSH Terms] OR “Recovery Room” [MeSH Terms]	122,244
#9	“Postanesthesia” [Title/Abstract] OR “Recovery” [Title/Abstract] OR “Postoperative” [Title/Abstract] OR “PACU” [Title/Abstract] OR “Post anesthesia care unit” [Title/Abstract] OR “Immediate postoperative” [Title/Abstract] OR “Postoperative Period” [MeSH Terms] OR “Postoperative Care” [MeSH Terms] OR “Recovery Room” [MeSH Terms]	1,244,717
#10	(“nurs*” [Title/Abstract] OR (“Postanesthesia Nursing” [MeSH Terms] OR “Nurses” [MeSH Terms] OR “Nursing” [MeSH Terms])) AND (“capno*” [Title/Abstract] OR “respiratory monitoring” [Title/Abstract] OR “Carbon Dioxide” [Title/Abstract] OR “end tidal carbon dioxide” [Title/Abstract] OR “respiratory assessment” [Title/Abstract] OR “respiratory complications” [Title/Abstract] OR (“Capnography” [MeSH Terms] OR “blood gas monitoring, transcutaneous” [MeSH Terms] OR “Carbon Dioxide” [MeSH Terms] OR “Pulmonary Ventilation” [MeSH Terms] OR “signs and symptoms, respiratory” [MeSH Terms])) AND (“Postanesthesia” [Title/Abstract] OR “Recovery” [Title/Abstract] OR “Postoperative” [Title/Abstract] OR “PACU” [Title/Abstract] OR “Post anesthesia care unit” [Title/Abstract] OR “Immediate postoperative” [Title/Abstract] OR (“Postoperative Period” [MeSH Terms] OR “Postoperative Care” [MeSH Terms] OR “Recovery Room” [MeSH Terms]))	277
#11	#10 FILTERS: English, Portuguese, Spanish	255
#12	#10 FILTERS: English, Portuguese, Spanish; Adult: 19+ years	118

**Table A2:** Search strategy—CINAHL (EBSCOhost)—search performed on 12 January 2024.

Search	Search Terms	Results
S1	TI nurs* OR AB nurs*	624,190
S2	(MH “Nurses+”) OR (MH “Perianesthesia Nursing”) OR (MH “Perioperative Nursing”)	254,721
S3	S1 OR S2	719,008
S4	TI capno* OR AB capno* OR TI “respiratory monitoring” OR AB “Respiratory monitoring” OR TI “carbon dioxide” OR AB “carbon dioxide” OR TI “end tidal carbon dioxide” OR AB “end tidal carbon dioxide” OR TI “respiratory assessment” OR AB “respiratory assessment” OR TI “respiratory complications” OR AB “respiratory complications”	9641
S5	(MH “Capnography”) OR (MH “Carbon Dioxide”) OR (MH “Signs and Symptoms, Respiratory+”) OR (MH “Blood Gas Monitoring, Transcutaneous”)	44,729
S6	S4 OR S5	50,121
S7	TI “postanesthesia” OR AB “postanesthesia” OR TI “recovery” OR AB “recovery” OR TI “postoperative” OR AB “postoperative” OR TI PACU OR AB PACU OR TI “post anesthesia care unit” OR AB “post anesthesia care unit” OR TI “immediate postoperative” OR AB “immediate postoperative”	212,894
S8	(MH “Post Anesthesia Care Units”) OR (MH “Post Anesthesia Care”) OR (MH “Postoperative Care”) OR (MH “Anesthesia Recovery”) OR (MH “Postoperative Period”)	42,008
S9	S7 OR S8	234,949
S10	S3 AND S6 AND S9	205
S11	S10 (Limited to English, Portuguese, and Spanish languages)	197
S12	S10 (Limited to English, Portuguese, and Spanish languages; age group: all adults)	74

**Table A3:** Search strategy—RCAAP—search performed on 20 January 2024.

Search	Search Terms	Results
1	capnografia AND enfermagem	5

## Appendix B. Data Extraction from the Studies

**Table A4:** Data extraction from the studies included in the scoping review.

Study	Reference Number, First Author’s Surname, Year of Publication, Country	Type of Study	Objective(s)	Population	Context	Concept (Barriers and Facilitators to the Use of Capnography for Respiratory Monitoring by Nurses in Phase I PACU)
S1	[26] Hutchison et al. (2008), United States of America (USA)	Randomized prospective study	Determine whether capnography used in isolation is more sensitive than pulse oximetry (with assessment of respiratory rate through observation or auscultation)	54 adult patients following orthopedic surgery, monitored for respiratory depression using capnography by nurses	PACU and general nursing care unit	**Barriers:** - Capnography requires more time from professionals to be implemented; - Nurses perceived greater difficulty in patient adherence due to limitations in daily activities, such as walking in the postoperative period. **Facilitators:** - Educating patients to facilitate their adherence to monitoring (for example, about the importance of the cannula and the meaning of the alarms); - The alarm produced by the capnography monitor led to a quicker response from nurses; - Allowed early identification of changes in respiratory function.
S2	[27] McCarter et al. (2008), USA	Quantitative, descriptive, cross-sectional study	Evaluate the effectiveness of monitoring in the postoperative period in patients with opioid PCA	634 adult postoperative patients receiving PCA with opioids, monitored with capnography by nurses	Postoperative period (Phase I and Phase II)	**Facilitators:** - Increased nurse awareness of respiratory changes; - Increased effectiveness of nursing interventions to minimize respiratory discomfort related to PCA; - Increased nurse confidence due to effective monitoring; - Quick response from the team to prevent serious consequences; - Increased perceived patient safety during this period.
S3	[28] Lakdawala et al. (2017), USA	Quality improvement project	Assess patients using the STOP-Bang screening tool; Compare high-risk and low-risk groups with respect to respiratory complications; Use and evaluate capnography in the postoperative period; Evaluate nurses’ perception of the OSA care protocol; Assess patient satisfaction with the OSA care protocol.	161 adult neuro-surgical patients screened for OSA using STOP-Bang, monitored with capnography by nurses	Preoperative unit, PACU, and neuro-surgery unit	**Barriers:** - Nurses observed greater patient resistance to adherence to capnography devices due to discomfort caused by the cannula and the nuisance of the alarm; - Nurses needed more knowledge about problem-solving methods related to the use of capnography in patients with OSA, to improve interpretation skills and response to each situation, as well as the indications for capnography and monitoring techniques. **Facilitators:** - Capnography proved to identify early signs of respiratory depression (such as apnea and snoring episodes), allowing for quick intervention; - Need to educate patients on the importance of using the device to ensure safety, raising awareness about the significance of monitoring, promoting adherence.
S4	[29] Jungquist et al. (2019), USA	Prospective observational study	Explore the effectiveness of using pulse oximetry, capnography, and minute ventilation to identify and anticipate opioid-induced respiratory depression in the post-anesthesia period	60 adult patients in PACU after spine, neck, hip, or knee surgery, monitored by nurses for opioid-induced respiratory depression	PACU	**Barriers:** - Nurses’ perception of patient non-adherence, related to discomfort, mask removal for nursing care, and other activities like eating and speaking; - Need for nurse training to instigate a change in the monitoring paradigm, reflecting limited knowledge of the technique and indications for capnography.
S5	[30] Scully (2019), USA	Quality improvement project (with mixed method)	Identify undiagnosed and high-risk patients with OSA in the preoperative period using the STOP-Bang screening tool; Train PACU nurses to recognize hypoventilation through capnography and intervene to prevent respiratory complications; Implement Practice Recommendation number 10 from the American Society of PeriAnesthesia Nurses (screening for OSA and monitoring of etCO2 in patients with OSA)	314 adult patients diagnosed with OSA, monitored by multidisciplinary team	PACU	**Facilitators:** - Training for professionals (introduction to capnography and monitoring); - Increased confidence in using capnography helped stimulate critical thinking (by applying it to other patients beyond the OSA population); - Improvement in care quality and patient safety are driving forces behind change; - Regular communication of results (via audit) motivated the team and promoted the implementation process; - Promoting sustainability by involving training and raising nurse awareness to provide the best care, integrating capnography monitoring into clinical practice; - Allowed the nurse to intervene before respiratory complications occurred.
S6	[31] Atherton et al. (2022), USA	Quantitative, descriptive-correlational, and longitudinal study	Evaluate the effectiveness of an educational program on ventilatory patterns using devices that assess carbon dioxide levels in postoperative patients	176 nurses	PACU	**Facilitators:** - After the educational program, nurses reported greater confidence and consequent security in using etCO2 and transcutaneous carbon dioxide monitoring and interpreting the values; - Prior knowledge and training with etCO2 monitoring.
S7	[21] Potvin et al. (2022), France	Randomized, controlled, prospective study	Study the rate of patients with alveolar hypoventilation before tracheal extubation or removal of the laryngeal mask through continuous capnography monitoring in the PACU	52 adult patients with endotracheal tube or laryngeal mask, monitored by nurses	PACU	**Facilitators:** - Nurse training facilitated the accurate interpretation of capnography results and the overall layout of monitoring systems; - Training should be coupled with a standardized respiratory monitory protocol to ensure effective use of capnography; - Standard monitoring, together with capnography, can improve patient safety by providing a comprehensive assessment of respiratory function, allowing early detection of respiratory complications.
